# MINSTED fluorescence localization and nanoscopy

**DOI:** 10.1038/s41566-021-00774-2

**Published:** 2021-03-15

**Authors:** Michael Weber, Marcel Leutenegger, Stefan Stoldt, Stefan Jakobs, Tiberiu S. Mihaila, Alexey N. Butkevich, Stefan W. Hell

**Affiliations:** 1 Department of NanoBiophotonics, Max Planck Institute for Biophysical Chemistry, Göttingen, Germany; 2 Clinic of Neurology, University Medical Center Göttingen, Göttingen, Germany; 3 Department of Optical Nanoscopy, Max Planck Institute for Medical Research, Heidelberg, Germany

## Abstract

We introduce MINSTED, a fluorophore localization and super-resolution microscopy concept based on stimulated emission depletion (STED) that provides spatial precision and resolution down to the molecular scale. In MINSTED, the intensity minimum of the STED doughnut, and hence the point of minimal STED, serves as a movable reference coordinate for fluorophore localization. As the STED rate, the background and the required number of fluorescence detections are low compared with most other STED microscopy and localization methods, MINSTED entails substantially less fluorophore bleaching. In our implementation, 200-1,000 detections per fluorophore provide a localization precision of 1-3nm in standard deviation, which in conjunction with independent single fluorophore switching translates to a -100-fold improvement in far-field microscopy resolution over the diffraction limit. The performance of MINSTED nanoscopy is demonstrated by imaging the distribution of Mic60 proteins in the mitochondrial inner membrane of human cells.

To resolve fluorophores that are far closer than the diffraction limit, all lens-based fluorescence nanoscopy methods have to make adjacent fluorophores discernible during registration and identify their coordinates with high precision. The elegance of STED microscopy^1,2^ derives from the fact that both tasks are performed in one go by the doughnut-shaped STED beam. By confining the fluorescence ability to a sub-diffraction-sized region around its central minimum, the STED doughnut beam both singles out the fluorophores that happen to be located in this region and establishes their position. The fluorescence ability and therefore the region defined by the STED doughnut are well described by the effective point-spread-function (E-PSF) of the STED microscope^3^, a Gaussian of full-width-half-maximum (FWHM) $d\approx \lambda /\left(2\text{NA}\sqrt{{1+\text{I}/\text{I}}_{\text{s}}}\right).$. Here *λ*, NA, *I* and *I_s_* denote the wavelength of the STED beam, the numerical aperture of the lens, the focal peak intensity at the doughnut crest and the intensity that reduces the fluorescence ability by half, respectively. Thus, scanning the sample with co-aligned (typically sub-nanosecond pulsed) excitation and STED beams separates fluorophores that are further apart than *d* and also locates them with the standard deviation *σ_E_* ≈ 0.42*d*.

Interestingly, if *d* becomes as small as the fluorophore itself (1–2nm), which is theoretically possible for *I >* 10^4^ *I*_s_, all fluorophores will be prevented from fluorescing except the one that happens to be located right at the central doughnut minimum. At this conceptual limit without background, detecting just a single photon per fluorophore renders a perfect image, because a single detection within a given time span verifies the presence of a fluorophore at a coordinate perfectly defined by the doughnut. No other super-resolution fluorescence concept can make emitted photons as informative as STED microscopy and its close derivatives^4^.

Unfortunately, separating and locating the emitters in one go comes at a cost. Since fluorescence blocking by STED typically entails intensities of *I*_s_ ≈ 1-10 MW cm^-2^, discerning fluorophores closer than *d* = 20 nm requires *I>* 100*I*_s_ ≈ 0.1-1 GW cm^-2^. Apart from the fact that applying such intensities to excited fluorophores promotes bleaching, doughnut minima are rarely –0.01*I* in practice^3^ due to residual alignment errors and aberrations. For *I >* 100*I*_s_, this means that the intensity at the minimum exceeds *I*_s_, which also degrades the fluorescence probability at the targeted coordinate and thus the fluorophore separation at distances well below 20 nm.

Here we introduce MINSTED nanoscopy, a STED-based super-resolution fluorescence microscopy method that can provide molecule-size (1–3nm) spatial resolution. This breakthrough has become possible by not requiring the STED doughnut to separate fluorophores (at small distances); its role is rather to establish the fluorophore’s position. Although we give up some of the elegance of the original STED concept, we obtain a fluorescence microscopy method whose resolution can be tuned from the diffraction limit down to the size of the fluorophores themselves. Compared with most other advanced STED and super-resolution methods^4^, MINSTED nanoscopy and the pertinent MINSTED localization entail less bleaching and reach the molecular scale with much fewer detected photons than achieved by popular camera-based techniques.

## Results

### MINSTED principle

To separate fluorophores at nanometre distances, MINSTED nanoscopy employs fluorophores that are transferred from an inactive (off) to an active (on) state and back. In the active state the fluorophore can be optically excited and de-excited by stimulated emission as in the concept called protected STED^5^. However, in MINSTED nanoscopy only one fluorophore within a diffraction-limited region is switched on at any given time, meaning that its coordinate is initially unknown across diffraction length scales^6,7^. The subsequent localization with the STED beam is greatly facilitated by the fact that the central minimum of the doughnut defines a coordinate to which the unknown coordinate of the fluorophore can be related. In the subsequent text, we refer to the position of the doughnut minimum as the ‘doughnut position’. Since it can be steered with beam deflectors at sub-nanometre precision, the doughnut position can be used for finding the position of the fluorophore in a sample: the closer it is to the fluorophore, the lower is the STED probability and the more probable is fluorescent emission. Evidently, the doughnut position entailing minimal STED must be identical with the fluorophore coordinate, hence the name MINSTED.

In contrast to the related concept called MINFLUX^8^, searching for the doughnut position with minimal STED is tantamount to searching for the position where the fluorescence is maximal. Yet this does not imply maximizing emission per se. First, the absolute emission rate is freely adjustable via the excitation beam power. Second, and more importantly, placing the doughnut minimum on top of the fluorophore to maximize the emission is neither required nor desired. Since the E-PSF is a Gaussian function, moving the E-PSF maximum in close proximity to the fluorophore does not provide the most precise localization per number of detected photons^8,9^. To find the peak of a Gaussian E-PSF it is in principle more photon-efficient to shift the peak aside and detect the rare, and hence more position-informative, photons generated at the Gaussian tail^8,9^. Unfortunately, the fluorescence photons from the tail are usually covered by the background signal, rendering localization with diffraction-limited Gaussian excitation beams unattractive for most applications. In MINSTED, however, we narrow the E-PSF down, leave the diffraction limit behind and make all detected photons more informative in general. While a ‘STED microscopy of *d* = 1 nm’ is still hard to reach with normal fluorophores, the localization precision *σ* continues to scale with $d/\sqrt{N}$. The number of detected photons *N* needed for reaching a certain *σ* decreases quadratically with decreasing *d*. Inserting the expression for *d* actually shows that *σ* ∝ $1/\sqrt{NI}$. Thus, MINSTED can shift the demand for many photons from *N* to *I*, that is from the photon-poor fluorescence to the photon-rich doughnut beam. Only those fluorescence photons that indicate the position of the fluorophore with respect to that of the doughnut are required.

Importantly, whilst making the doughnut more intense and zooming in on the fluorophore position, the doughnut can be translated so that the fluorophore always experiences intensities of the order of *I*_s_ and avoids the intensities *I≫ I_s_* that are found around the doughnut crest^10,11^. As we show in this paper, the unique combination of all these factors bestows MINSTED nanoscopy with molecule-size precision and resolution.

### MINSTED implementation, localization algorithm and simulations

**Fig. 1:**
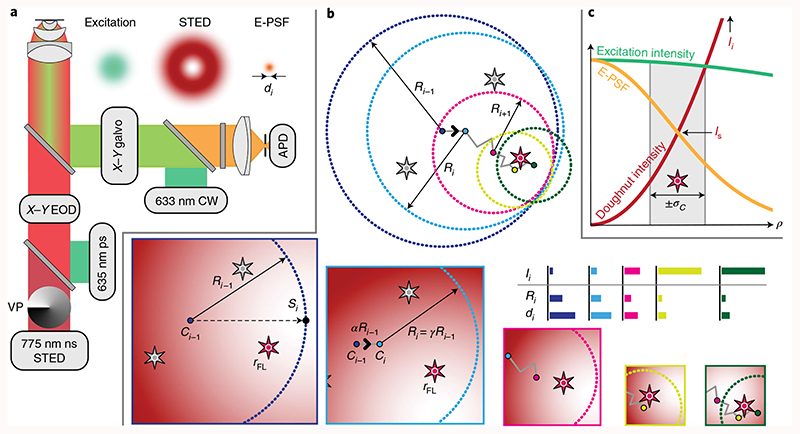
Principles of MINSTED localization. **a**, STED setup with co-aligned pulsed lasers for excitation and STED at 635 and 775 nm, respectively, and a vortex phase plate (VP) for helical phase modulation converting the STED beam into a doughnut; the inserts sketch the excitation and STED probability in the lens focal plane, along with that of the fluorescence (E-PSF). The 633 nm CW laser was used for fluorophore pre-identification in the focal plane, while the *X-Y* galvo unit also maintained the optical conjugation of the confocal avalanche photodiode (APD) detector to the centre *Cį* of the circular scan performed by the electro-optical lateral deflector (*X*-*Y* EOD). **b**, The active fluorophore (red among grey stars), located at unknown position *r*_FL_, was localized by circular *X*-*Y* scans. For each photon detection *i*, the centre *C_i_* was shifted by a fraction *α* of the radius *R_i_* toward the doughnut minimum *S_i_*. Simultaneously, *R_i_* and the FWHM *d_i_* of the E-PSF were scaled by *γ*<1. The centre *C_i_* thus converges to the fluorophore position (grey line) as indicated in the lower panels that also sketch relevant parts of the doughnut for some detections during the homing-in process. Once a minimum radius *R*_min_ (yellow) is reached, only *C_i_* is updated and the localization terminated after the fluorophore becomes inactive (*N* detections). The column diagrams illustrate the decrease of *R_i_* and of *d_i_* with increasing doughnut intensity *I_i_*. **c**, Normalized probability of excitation (green) and fluorescence detection (E-PSF, yellow) as a function of radial distance *ρ* from the focal point, along with a non-normalized intensity profile of the STED beam doughnut (red). Although *I_i_*, is constantly increased during the localization to sharpen the E-PSF, the intensity experienced by the fluorophore remains about *I*_s_ within the *±σ_C_* position range of the centre positions *C_i_* highlighted in grey.

We implemented MINSTED in a confocal scanning microscope with electro-optic deflectors (EODs) and galvanometer mirrors for fast and slow scanning in the focal plane, respectively (Fig. 1a). After identifying an individual fluorophore by scanning fleetingly over the sample and estimating its position with 5–10 detections, the co-aligned excitation and STED beams were circled around a position estimate *C_i_* with a radius *R_i_*≈ *d_i_*/2. Both *C_i_* and *R_i_* were updated after each photon detection *i* (Fig. 1b). Starting at *i* = 0 with a diffraction-limited E-PSF diameter *d*_0_ given for *I=*0, the scan centre *C_i_* was shifted by a fraction *α* of *R_i_* toward the doughnut position, that is the E-PSF maximum, when detecting the next photon (*i* + 1). In other words, the doughnut minimum was moved tentatively closer to the fluorophore. This measure allowed us to sharpen the E-PSF by increasing the doughnut intensity *I_i_* and reduce *R_i_* by a factor *γ* at the same time, so that the ensuing smaller *d_i_* left the ratio *R_i_*/*d_i_* essentially unchanged. Therefore, despite the progressively higher *I_i_* and the steeper E-PSF slope, the fluorophore constantly experienced moderate doughnut intensities in the ballpark of *I*_s_ (Fig. 1c). A reduction of 3% per photon detection (*γ*=0.97) of *d_i_* and a step size of 15% of the scan radius (*α* = 0.15) were typically used. We also set a limit on the minimal radius *R*_min_ and on the highest doughnut intensity *I*_max_. During the circular scanning, the synchronously steered galvanometer mirrors ensured that the scan centre *C_i_*, that is the position estimate of the fluorophore, remained projected onto the confocal detector. As we zoomed in on the fluorophore, the precision *σ* improved with decreasing *d_i_* whilst the average emission rate remained largely constant.

**Fig. 2:**
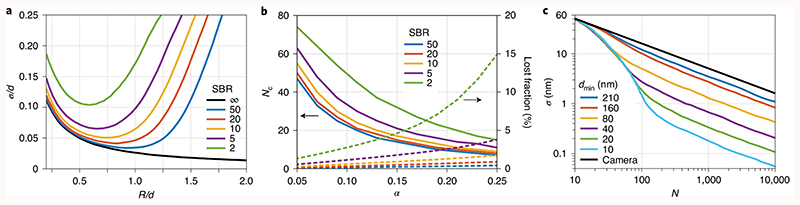
Simulation of MINSTED localization with *N* = 100 detected photons. **a**, Localization precision *σ* with different ratios of scan radius *R* to FWHM *d* of the STED microscope’s Gaussian E-PSF with the SBR as the parameter. While the hypothetical infinite SBR case calls for *R* maximization (black line), the presence of the background enforces O.5*d* ≤ *R* ≤ *d*. For large *R* the information provided by the detection of a single photon is masked by the background, whereas for a small *R* it is masked by the many other photon detections connected with an E-PSF maximum of finite *d*. In the localization process, the values of *R* and *d* are updated for every photon count *i* to the specific values *R_i_* and *d_i_*, respectively. **b**, Detections *N*_c_ necessary until the distribution of scan centre positions *C_i_* converges to a final distribution (with static *d*); percentage of simulations with centre positions *C_i_* further than *d* away from the fluorophore and hence classified as lost. **c**, Localization precision *σ* as a function of total number of detections *N* with *d*_min_ as the parameter.

To assess the optimal ratio *R_i_/d_i_*, we simulated the precision *σ* expected for different *R_i_/d_i_*, intensity steps *I_i_* and peak signal-to-background ratios (SBRs) (Fig. 2). The SBR is a crucial parameter defined as the maximum detection rate from a fluorophore divided by a uniform detection rate in the sample. For zero background and a given number of detections *N*, a Gaussian E-PSF achieves a higher precision when *R_i_*/*d_i_* is large. However, in the practical range 5 < SBR < 50 and for *N* = 100 detections, *R_i_*/*d_i_* = 0.5 is a better choice (Fig. 2a) because in the presence of the background the value of *σ* becomes smaller when the emitter is closer to the E-PSF maximum and provides more photons. The step size *α* has several effects on the distribution of the centre positions *C_i_* and hence on the position estimate. A small *α* increases the *N* needed to reduce the distance between *C_i_* and the fluorophore, and to converge to a final centre distribution (Fig. 2b). A larger *α* helps to approach the fluorophore quickly, but the weaker correlation amongst the successive *C_i_* positions bears the risk of not converging at a low SBR. Furthermore, the reduction of *d_i_* and *R_i_* by a factor *γ* is tightly connected to the best step size *α*. Making *α* larger implies that 1 – *γ* can also be larger than 3%. Increasing *α* and decreasing *γ* entails that the number *N*_c_ of detections needed to reach the final *d*_min_ becomes smaller. For a low SBR the risk of ultimately missing the fluorophore position grows, as expected (Fig. 2b; see also Supplementary Figs. 1–4 and Supplementary Video 1).

Altogether, a simulation of 500 localizations as a function of a finite number of detections *N* showed that the chosen parameters should provide a robust and precise localization. As the doughnut scan centre homes in on the emitter, the *C_i_* series serves as the permanently updated position estimate of the emitter. After reaching *d*_min_ at *i* = *N*_c_ and until reaching *i* = *N*, the coordinate average ${\bar{C}}_{N}=C_{N_{c}\leq i\leq N}$ was our localization result. The simulation reveals the importance of the counts up to about *N*_c_ for zooming in and reducing the *N* required for a certain *σ*. It also confirms that *σ* scales with $d_{\min}/\sqrt{N-N_{\text{c}}+1}$ (Fig. 2c). Concretely, *d*_min_ = 40 nm is predicted to yield *σ ≈* 3 nm with only *N* = 100 photon detections.

### Experimental MINSTED localization precision

**Fig. 3:**
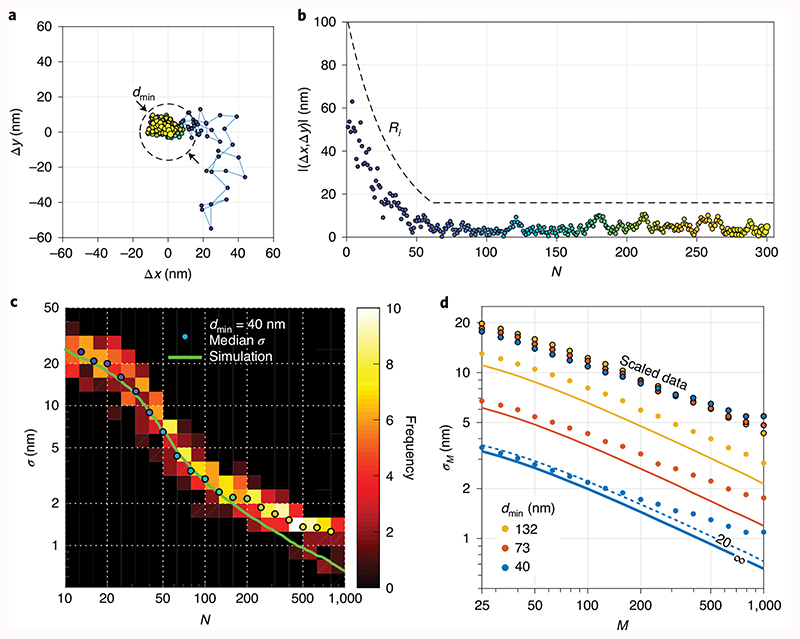
MINSTED localization of single fluorophores. **a**, Localization trace from the first *i* = 1 (blue) to the last detection *i* = 300 (yellow) with the final scan circle (dashed line) around the estimated *(x,y)* position. **b**, Scan radius *R_i_* (dashed line), distance (Δ*x*,Δ*y*) from the final estimated position to the scan centre *C_i_* (points) from *i* = 1 (blue) to *i* = 300 (yellow) detections. **c**, Histogram of precision *σ* of grouped localization traces and their median *σ* showing good agreement with simulation. **d**, Measured precision *σ_M_* (derived from segments of *M* detections measured after *d*_min_ had been reached) showing how the increase in STED doughnut power improves the precision in linear proportion to *d*_min_, which is also confirmed by the overlap of data points when all points are scaled to *d*_min_ = 200 nm for comparison. Solid lines show simulation results for SBR = ∞, the dashed line for SBR = 20 as indicated. Note the logarithmic display in **c** and **d**.

To test these predictions, we localized immobilized individual Atto 647N fluorophores on coverslips^12^ using MINSTED with *d*_min_ ≈ 40 nm. Driven by each detection *i*, the scan centre progressed toward the fluorophore and ultimately meandered around the estimated final coordinate (Fig. 3a,b and Supplementary Video 2). Recording many of these traces for many fluorophores allowed us to explore the attainable precision. The fluorophores were localized multiple times and the localization precision was analysed between the different localizations of the same molecule. To attribute localizations to individual fluorophores, we clustered localizations that were ≤25 nm apart. Only sets with more than five localizations were analysed and the scan centres *C_i_* were regarded as the fluorophore coordinate estimates for *N* < *N_c_*, as in the simulations. Once *d*_min_ = 40 nm was reached, the ‘meandering’ positions *C_i_* were averaged to ${\bar{C}}_{i}$ until the specified *N* and hence ${\bar{C}}_{N}$ was reached. Within each localization cluster, the estimated final coordinates were calculated at multiple photon numbers to establish *σ* as a function of *N*.

Our experiments show that *σ* decreases rapidly with decreasing *d_i_* until *d*_min_ is achieved at *N*_c_ ≈ 60 (Fig. 3c). For *N> N_c_*, the precision *σ* follows the $1/\sqrt{N-N_{c}+1}$ dependence (compare Fig. 2c) until it deviates from the simulation at about *σ* < 2 nm. This deviation is likely due to residual drifts of the fluorophore and/or the setup. The measured *σ* at around *N* = 10 is slightly better than the previously simulated values, because the 5–10 detections gained from the initial fluorophore identification by galvo-scanning provided *σ*_0_ ≈ 60 nm right at the outset. Consideration of this *σ*_0_ resulted in an excellent agreement between the simulated and experimental *σ* as a function of *N* (Fig. 3c). Since we cannot exclude residual movement of fluorophores on distances substantially less than the standard deviation *σ*_C_ of *C_i_*, we can safely assert that in our experiments MINSTED reached *σ* = 2–3 nm with just *N* = 200 detections.

Next, we measured *σ* obtained after *d*_min_ had been reached. Since the total number of detections before bleaching typically exceeded 1,000 per fluorophore, we split the resultant *C_i_* traces into segments of different sizes *M* and calculated the standard deviations *σ_M_* of the localization in these segments. To avoid boundary artefacts, we explored the range *N* – *N_c_* > 25. In agreement with the simulations, the measurements again followed the $1/\sqrt{N-N_{c}+1}$ relation and the linear dependence on *d*_min_ (Fig. 3d and Supplementary Fig. 5). To highlight the latter, we also scaled the measured *σ_M_* to *d*_min_ = 200 nm so that any difference from the linear dependence could be noticed in the overlay.

At *σ* < 3 nm, the measured *σ* deviates from the simulations as before. However, the data show that at *d*_min_=40 nm, 1,000 detected photons yield molecule-size precisions *σ* ≈ 1 nm. If residual movements of the stage or the fluorophore could be avoided, ~500 detections at SBR = 20 would suffice for *σ* ≤ 1 nm. Indeed, comparison of the measured precision with that simulated for the ideal SBR = ∞ case shows improved agreement for smaller *d*_min_, indicating that the STED doughnut not only improves the information of the detected photons by confining their origin in space, but also by suppressing the background.

### MINSTED fluorescence nanoscopy in cells

The separation of emitters in MINSTED nanoscopy requires fluorophores that can be transferred from a lasting state that is non-responsive to excitation light into a semi-stable state leading to fluorescence upon excitation. Silicon rhodamine (SiR) fluorophores with two unsubstituted photoactivatable *ortho*-nitrobenzyl (ONB) caging groups proved suitable for MINSTED because photoactivation at the 355 nm wavelength activated the SiR fluorophores enabling STED at a wavelength of 775 nm with no concurrent two-photon activation^13^.

To demonstrate its potential for biological imaging, we used MINSTED nanoscopy to image the mitochondrial inner membrane protein Mic60 in chemically fixed human cells^14^. The mitochondrial inner membrane folds into cristae, large membrane invaginations that increase the surface area of this membrane. Mic60 is enriched at crista junctions^15^, which are round or slit-like structures that connect the crista membranes with the mitochondrial inner boundary membrane that is parallel to the mitochondrial outer membrane.

**Fig. 4:**
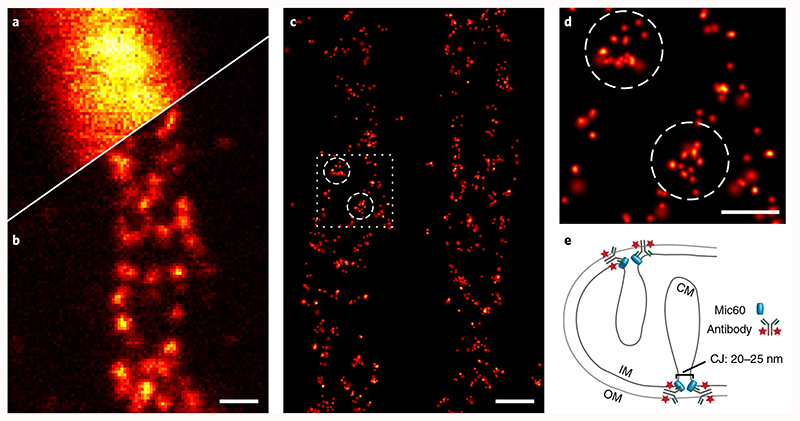
MINSTED nanoscopy of mitochondrial protein Mic60. **a**,**b** Confocal (**a**) and STED (**b**) images with *d ≈* 60nm of the same mitochondrion taken after simultaneous activation of all fluorophores. **c**, MINSTED nanoscopy image of similar mitochondria resolving the Mic60 clusters (3,607 localizations acquired in 33min, 1,766 localizations with *N* – *N_c_≥* 200 detections and *d*_min_ = 54nm). **d**, Excerpts of data as indicated in **c**. **e**, Schematic of the presumed localization of Mic60 in the mitochondrial inner membrane. IM, inner membrane; OM, outer membrane; CM, crista membrane; CJ, crista junction. Scale bars: **a**-**c**, 200 nm; **d**, 100 nm.

Immunolabelling of cultured human U-2 OS cells with ONB-2SiR-labelled primary anti-Mic60 antibodies allowed us to compare MINSTED nanoscopy with confocal and STED images recorded after activation of ONB-2SiR (Fig. 4). Confocal microscopy was unable to provide details of the distribution of Mic60 in the mitochondria (Fig. 4a). Featuring a resolution of about 60 nm, the recorded STED images demonstrated a clustering of Mic60, but failed to resolve individual emitters (Fig. 4b). By contrast, MINSTED accomplished this feat (Fig. 4c,d), recording 1.8–2.4 raw localizations per second and resolving individual fluorophores with a median precision of *σ* = 2.1 nm (Supplementary Fig. 6).

Figure 4c,d was reconstructed from 49% of the raw localizations; 48% of the raw localizations were dropped because they were too dim (*N* – *N*_c_ < 250) and a further 3% were dropped because they showed an excessive *σ*, that is they did not converge. We observed that consecutive multiple localizations of the same fluorophore were limited to a few percent of all localizations: 56% of the *raw* localizations were observed within two scans of the image area from the previous localization, but only 4.7% of those were located within less than 20 nm distance. Although 6.3% of the filtered localizations were observed within two scans and were within 20 nm of the previous localization, only 1% fell within the <2 nm distance. Hence, we can conclude that only a few fluorophores were localized and rendered multiple times.

For this study, we relied on primary antibodies that were labelled by azide modification of the glycans on the antibody heavy chain, so that the distance between the antibody binding site and the fluorophore was as small as 6–10 nm (PDB ID code: 1HZH (ref. ^16^)). The localization precision of individual fluorophores was three times higher than this distance, highlighting the limits set by the labels on extracting biological information at the single-digit nanoscale. Since fluorescence microscopy cannot reveal anything but the fluorophores in the sample, our results show that MINSTED reaches the conceptual limits of this imaging modality. Nevertheless, our two-dimensional MINSTED data provide valuable insights about the nanoscale distribution of Mic60 in mitochondria. We repeatedly recorded a circular arrangement of Mic60 in mitochondria using MINSTED (Fig. 4d), which is in excellent agreement with the current understanding of Mic60 forming small ring-like assemblies at cristae junctions^17^. With future three-dimensional implementations of MINSTED, complex structures such as those found in the mitochondrial inner membrane should be accessible in detail.

## Discussion

Under the provision that adjacent fluorophores are sequentially active and hence separable, MINSTED nanoscopy can deliver molecule-scale resolution like its MINFLUX counterpart^18^. However, in MINSTED a resolution of *σ* = 2 nm or *d* = 4.7 nm is attained with a total of just 200 detections, in close agreement with the simulations. The reason is that the STED doughnut suppresses spurious signal from the neighbourhood of the targeted fluorophore, rendering the MINSTED images (Fig. 4) almost background-free.

Another strength of the described MINSTED implementation is that the photon-by-photon update of the localization removes virtually all bias due to inaccurate assumptions on the background or doughnut shape. Furthermore, the unequivocal repositioning of the doughnut centre in the right direction allows for an aggressive reduction of *d_i_* and hence also of *N*. Once *d*_min_ is reached, each subsequent photon refines the scan centre *C_i_* and lowers the uncertainty on the position estimate ${\bar{C}}_{N}$. In fact, continuous updating of *C_i_* tracks the fluorophore until it bleaches or switches off. As it provides the most photon-efficient localization so far, MINSTED will also be useful for tracking rapidly moving emitters. Our MINSTED protocol can be further refined by dynamically adapting *R_i_*/*d_i_* in response to the background.

Besides, MINSTED is able to single out individual fluorophores if any other active fluorophore is at least *d_i_* + (1 + 2*±*) *R_i_* away; with our typical parameters this distance amounts to about 1.8*d_i_*. In contrast to MINFLUX, for MINSTED only a sub-diffraction region around the targeted fluorophore must remain free of other active fluorophores when *d*_min_ ≪ *d*_0_. Evidently, future MINSTED research will include multiple colour channels using spectrally shifted fluorophores, three-dimensional recordings using three-dimensional doughnuts and technically more sophisticated implementations with adaptable doughnut arrays or sets of standing waves (also known as structured illumination).

The selective spatial targeting of the doughnut minimum constitutes a fundamental difference from earlier applications of STED microscopy to single-fluorophore localization, whereby the doughnut is scanned laterally across the focal plane to map out the E-PSF centroid rendered by each fluorophore^12,19,20^. This established combination of single fluorophores and STED works reliably only for bleaching-resilient emitters, such as nitrogen-vacancy centres^19^, or for low doughnut intensities, because the intense doughnut crest usually bleaches the fluorophore before the whole E-PSF is acquired. Moreover, precise rendering of the E-PSF is typically compromised by the tendency of the fluorophores to blink. In MINSTED, although the doughnut intensity is constantly increased, bleaching and blinking aggravation is avoided. For attaining nanometre spatial resolution, MINSTED nanoscopy requires neither intensities of >10^4^ *I*_s_ nor doughnut minima of <1% since the on/off separation of spatially tight fluorophores is not performed by the doughnut but by the on/off switching of individual fluorophores. However, the doughnut brings about the advantage that it additionally assists the on/off separation at sub-diffraction length scales.

Seeking a fluorescence minimum, as in MINFLUX, has a conceptual advantage over localizing with a Gaussian E-PSF unless the background comes into play. When narrowing the search range by increasing the doughnut intensity, the excitation doughnut in MINFLUX^8^ is more prone to worsening background levels than is the STED doughnut in MINSTED. MINFLUX trades off the SBR for a smaller fluorophore-to-doughnut distance, as long as the deterioration in localization precision due to the lower SBR is overcompensated by the improvement gained from the smaller distance. In MINSTED, however, the fluorophore is exposed to nearly the same excitation and STED intensity throughout the process, irrespective of the fluorophore-to-doughnut distance. As a result, compared with MINFLUX, MINSTED experiences substantially reduced variations in the fluorescence signal and SBR. In fact, we found that higher STED doughnut intensities at 775 nm keep the background low, even for small *d*_min_ values. For this reason, MINSTED is currently on a par with or even outperforms MINFLUX in key aspects.

In most of our MINSTED imaging, *d*_min_ was not reduced below 40 nm because a higher STED beam power would have increasingly destabilized the system by heating. Requiring a STED beam is an added complexity of MINSTED compared with MINFLUX, but the precision *σ* values achievable with either of the two molecule-scale resolution approaches will ultimately depend on the background. In any case, by featuring excellent background suppression, MINSTED should become substantially faster and handle higher densities of fluorophores than most super-resolution methods in the future.

Finally, the introduction of MINSTED underscores that the idea of optically injecting a movable reference coordinate is transformative in the art of the localization of emitters. In conjunction with on/off state separation, MINSTED enlarges the scope of far-field fluorescence nanoscopy with molecule-size resolution, which, due to its 100-fold improvement over the diffraction limit, is poised to break new ground.

## Online content

Any methods, additional references, Nature Research reporting summaries, source data, extended data, supplementary information, acknowledgements, peer review information; details of author contributions and competing interests; and statements of data and code availability are available at https://doi.org/10.1038/s41566-021-00774-2.

## Methods

### MINSTED setup

The setup consists of an epi-fluorescence microscope with a dual-channel confocal laser scanning system using a Leica × 100/1.4NA oil-immersion objective lens. Two galvanometer mirrors and pupil relay optics allowed for rapid beam scanning over a quadratic sample area of about 100 μm extent *(x,y)*. A continuous-wave (CW) HeNe laser provided fluorescence excitation at the 633 nm wavelength for rapid overview. A single-photon counting module detected the fluorescence light in the 650–750 nm range. A confocal pinhole with a diameter of 0.5 Airy units blocked out-of-focus light. For STED microscopy and single-molecule localization, an additional illumination path without moving parts was implemented. Two electro-optic deflectors with pupil relay systems featured beam scanning within a square image area of about 2.6 μm extent. A 635 nm pulsed diode laser delivered excitation pulses of about 100 ps duration, whereas a 775 nm pulsed fibre laser provided STED pulses of about 1 ns duration. A vortex phase plate imprinted a 2π phase ramp on the phase front of the STED beam and a polarization controller converted it to circular polarization to shape the STED beam into a doughnut profile. A laser at 355 nm wavelength illuminated the STED image area to photoactivate the fluorophores. All laser beam powers were modulated with short response times of several microseconds. The sample was mounted on an *X-Y-Z-piezo* positioning stage whose position was locked by a sample-tracking system. For this purpose, the position of fiducial markers was monitored with infrared light from a super-luminescent light-emitting diode and fast CMOS cameras. The tracking system issued the closed-loop control signals to cancel the sample drift. The MINSTED microscope was fully controlled by an FPGA board and a custom control program. Our software ran diffraction-limited overview scans using only the galvanometer beam scanner as well as high-resolution STED image scans and single-molecule localizations using both scanners synchronously. For STED imaging and localization, a time gate blocked the early fluorescence detections during the STED pulses. A graphical user interface allowed definition of the measurement parameters and retrieval of the measurement results.

### Immobilization of Atto 647N fluorophores

Atto 647N molecules were sparsely distributed and immobilized on cover slides as described in ref. ^8^. A flow channel, consisting of a cleaned coverslip glued to a microscope slide with double-sided scotch tape, was rinsed with 100 μl phosphate-buffered saline (PBS; 137 mM NaCl, 2.7 mM KCl, pH 7.4). The channel was filled with 15 μl biotinylated bovine serum albumin (biotinylated BSA; A8549, Sigma Aldrich) 0.5 mg ml^-1^ in PBS. After 4 min of incubation, the channel was flushed with 100 μl PBS and filled with 15 μl streptavidin (11721666001, Sigma Aldrich) 0.5 mg ml^-1^ in PBS. After an incubation time of 4 min, the channel was flushed with 100 μl PBS and filled with 15 μl of 200 pM hybridized biotin-DNA/Atto647N-DNA in PBS^8^. After 4 min of incubation, the channel was flushed with 100 μl PBS and filled with 0.01% (w/v) poly-L-lysine (P8920, Sigma Aldrich) in PBS for 10 min. After flushing with 100 μl PBS, the channel was filled with 15 μl freshly diluted silica shelled silver nanoplates (SPSH1050, nanoComposix) 2.5 μg ml^-1^ in PBS. After 10 min of incubation the channel was flushed with PBS again, filled with 15 μl ROXS buffer^21^ and sealed with epoxy glue (Hysol, Locktite).

### Antibody conjugation

The labelling of the antibody using glycan modification and strain-promoted click chemistry, together with the synthesis of the dye used was as described previously^13^. In short, the rabbit monoclonal antibody (ab245764, Abcam) was modified with azide groups using a commercial enzyme system (GlyClick, Genovis). After the modification, 250 μg antibody in 200 μl Tris-buffered saline (TBS; 20 mM Tris HCl, 150 mM NaCl, pH 7.6) was mixed with 50 μl dimethylformamide containing 50 μg dibenzylcyclooctyne dye and stirred overnight. The free dye was removed via phase extraction by adding 600 μl distilled water, 90 μl saturated (NH4)2SO4 solution and 900 μl *tert*-butanol, vortexing and separating the phases after a short centrifugation pulse. The aqueous phase (about 600 μl) was diluted using 600 μl TBS. The labelled antibodies were aliquoted and stored at −20 °C.

### Cell labelling

The human osteosarcoma cell line U-2 OS was obtained from the European Collection of Authenticated Cell Cultures (ECACC; cat. no. 92022711, lot 17E015) and cultivated on coverslips in McCoy’s medium (Thermo Fisher Scientific) supplemented with 10% (v/v) fetal bovine serum (Thermo Fisher Scientific), 1% (v/v) sodium pyruvate (Sigma Aldrich) and penicillin–streptomycin (Sigma Aldrich). The cells were fixed using 8% (w/v) paraformaldehyde in PBS for 5 min, permeabilized with 0.5% (w/v) Triton X-100 for 5 min and quenched with 100 mM NH_4_Cl in PBS for 5 min. The fixed cells were washed with PBS, blocked with 2% (w/v) BSA in PBS and treated with the primary antibody in the same buffer for 1 h, washed with 2% (w/v) BSA in PBS, treated with a secondary goat anti-rabbit antibody conjugated with Alexa 647 as counterstain for MINSTED and washed with PBS. The cells were incubated with freshly diluted silica shelled silver nanoplates (SPSH1050, nanoComposix) 2.5 μg ml^-1^ in PBS for 10 min and washed with PBS again.

### Cell imaging

The confocal and STED images were recorded using a commercial Abberior Instruments Expert Line microscope equipped with a 775 nm 40 MHz STED laser and a 640 nm excitation laser after activation with a spectrally broad 405 nm light-emitting diode as described in ref. ^13^. For MINSTED, the labelled cells were incubated with freshly diluted silica shelled silver nanoplates in PBS for 20 min and then washed with PBS. The samples were mounted with buffer (20 mM HEPES, 150 mM NaCl, pH 7) using Twinsil (Picodent). Before MINSTED, the cells were selected based on the counterstain signal and the Alexa 647 dyes were bleached using low-power STED light. The localization routine was started without the excitation laser to equilibrate the temperature in the immersion oil and sample, which were warmed up by the STED laser. After 10 s, the excitation laser was enabled and the caged dyes were sparsely activated using 355 nm light when searching for another active fluorophore. Over the duration of the measurement, the ultraviolet laser power was slowly increased to keep the activation rate constant. The imaging was stopped when no further molecules could be activated.

### Data analysis

The localizations were analysed based on the centre positions *C*_*i* ≥ *N_c_*_ at *d*_min_. The localizations were further selected with a maximum filter on the standard deviation *σ_c_* of the *C_i_*, together with a minimum filter on the number of detected photons *N*. The precision of each localization was estimated as described in the Supplementary Information and validated by simulations (Supplementary Fig. 3). The image was rendered with the estimated precision lower-bounded to 3 nm.

### Reporting summary

Further information on research design is available in the Nature Research Reporting Summary linked to this article.

## Supplementary Material

Supplementary material

Supplementary video 1

Supplementary video 2

## Data Availability

The data that support the plots within this paper and other findings of this study are available from the corresponding author upon reasonable request. Sample data to generate Figs. 2b and 3a,b,d; Supplementary Figs. 1–3 and 5; and Supplementary Videos 1 and 2 is available in the supplementary archive.

## Code availability

The simulation code to generate Figs. 2b and 3a,b,d; Supplementary Fig. 1–3, 5; and Supplementary Videos 1 and 2 is available in the supplementary archive.
