# Supplementary material for: MINSTED fluorescence localization and nanoscopy
Source: Nat Photonics. Author manuscript; Available in PMC 2021 May 4. (PMC7610723; doi:10.1038/s41566-021-00774-2)
Supplement: Supplementary material [file EMS122729-supplement-Supplementary_material.pdf]

nature portfolio

https://doi.org/10.1038/s41566-021-00774-2

Supplementary information

MINSTED fluorescence localization and nanoscopy

In the format provided by the authors and unedited

Supplementary information

Supplementary text

MINSTED localization method

Image scans were performed until the total number of detected photons of the current and previous columns and rows exceeded a threshold n_{ON} of typically 5-10. When the threshold was exceeded, the image scan was interrupted and the detections at the last image positions were used for an initial estimation $\vec{r}_0$ of the fluorophore position $\vec{r}_{\mathrm{fl}}$ by a weighted average. A circular scan was started, whose initial centre position $\vec{C}_0 = \vec{r}_0$ and whose radius $R_0 \approx d_0/2$ was approximately half the PSF diameter as illustrated in Fig. 1b. During the localization, the centre converged towards the fluorophore position. Thus, the fluorophore was exposed to a moderate STED intensity because it was kept within a distance d from the doughnut position $\vec{S}$ most of the time, typically at about the scan radius R (Fig. 1c). Upon detection of photon i at the doughnut position $\vec{S}_i$, the centre, scan radius and E-PSF diameter were updated immediately. The centre was moved towards $\vec{S}_i$ by a fraction α and the scan radius and E-PSF diameter were scaled by $\gamma < 1$. The scanning continued around the new centre $\vec{C}_i = (1-\alpha)\vec{C}_{i-1} + \alpha\vec{S}_i$ with radius $R_i = \gamma R_{i-1}$ and $\mathrm{d}_i = \gamma\ \mathrm{d}_{i-1}$ until the next photon was detected and the update repeated. The scan radius and the E-PSF diameter were decreased only until reaching their preset lower limits, whereas the centre position was updated throughout. The localization was terminated if less than a minimum of n_{OFF} photons were detected within a time interval τ_{OFF}.

The real-time FPGA control logic was kept simple and lean. All localization traces consisting of centre positions, doughnut positions, detected photons and detection times were transferred to the computer for storage and further evaluation using MATLAB and custom analysis tools. The fluorophore position after N photon detections was estimated by the last centre position for $N \leq N_{\mathrm{c}}$, where N_{c} is the number of detections to reach the smallest scan radius and sharpest E-PSF. For $N > N_{\mathrm{c}}$ the fluorophore position was estimated by the average centre positions during the remaining photon detections.

$$\hat{\vec{r}}(N) = \begin{cases} \vec{C}_N & \forall\, N \leq N_{\mathrm{c}} \\ \dfrac{1}{N - N_{\mathrm{c}} + 1} \displaystyle\sum_{i=N_{\mathrm{c}}}^{N} \vec{C}_i & \forall\, N > N_{\mathrm{c}} \end{cases} \tag{S1}$$

MINSTED localization uncertainty

Due to the continuous update of the centre position, the localization uncertainty – this means the Cramer-Rao bound (CRB) – could not be easily obtained analytically for the interesting case of many photon detections. Instead, we repeatedly simulated the localization of a fluorophore and estimated the localization uncertainty by the root mean square (RMS) localization error versus the number of detected photons N.

Given the estimates $\hat{\vec{r}}_j(N) = \left(\hat{x}_j(N), \hat{y}_j(N)\right)$ of the fluorophore position after N detections for $j = 1 \dots K$ runs of the simulation, the localization uncertainty along x and y was estimated by the root mean square errors along the coordinate axes:

$$\sigma_{\mathrm{loc}}^2(N) = \frac{1}{K}\left(\sum_{j=1}^{K}\left(\hat{x}_j(N) - x_{\mathrm{fl}}\right)^2 \sum_{j=1}^{K}\left(\hat{y}_j(N) - y_{\mathrm{fl}}\right)^2\right)^{1/2} \tag{S2}$$

Simulated localizations that ended prematurely after $i < N$ detections (too few detections for too long terminate the localization) or whose positions were off by more than five times the median error of the simulations were flagged as failures for the remaining detections. Equation $(\mathrm{S2})$ was evaluated for the successful localizations only. Fig. 2a–c, Fig. 3c,d illustrate localization uncertainties with simplified fast calculations, whereas Suppl. Fig. S1 and Suppl. Fig. S2 illustrate results based on complete simulations including the detection times and the termination of the fluorophore's active state.

For the experimental localizations, the true fluorophore position and thus the accuracy is unknown. Based on simulated localizations, we found that the estimation of the localization precision similar to camera-based localizations is unreliable due to the history of the centre trace, which introduces a varying degree of correlation among the centre positions. Instead, we estimated the precision of an individual localization by sub-sampling. Therefore, we split the centre trace in groups of different sizes M, estimated the fluorophore position for each of these groups, and extrapolated the precision from the groups to the entire localization.

Given $N \gg N_{\mathrm{c}}$ detected photons, we calculated for consecutive groups of $M = \left[10^{\{1.5,1.6,1.7,\dots\}}\right] \leq (N - N_{\mathrm{c}} + 1)/5$ detections the standard deviations $\sigma_M(M)$ of the groups' mean centre positions. We then estimated the precision of the localization by extrapolating the relation

$$\sigma_M(M) \approx \frac{\hat{\sigma}_1}{\sqrt{M}} + \hat{\sigma}_\infty \tag{S3}$$

to $M = N - N_{\mathrm{c}} + 1$. Here, $\hat{\sigma}_1 > 0$ is approximately the standard deviation among uncorrelated centre positions and $\hat{\sigma}_\infty \geq 0$ is an empirical offset, both obtained by least-squares decomposition of $\sigma_M(M)$.

$$\hat{\sigma} \approx \frac{\hat{\sigma}_1}{\sqrt{N - N_{\mathrm{c}} + 1}} + \hat{\sigma}_\infty \tag{S4}$$

Suppl. Fig. S3 compares the localization uncertainty obtained from numerous simulated localizations with the estimated precision extracted from the individual localizations. The extracted precision provides a reasonable and rather conservative estimate of the true uncertainty, but at least 200 detected photons are required for this extrapolation.

Simulated localization

The fluorophore was placed at $\vec{r}_{\mathrm{fl}} = (x_{\mathrm{fl}}, y_{\mathrm{fl}})$ and the localization started with a centre position $\vec{C}_0$ that was normally distributed in the (x, y) plane around this position with a standard deviation 90 nm. Hence, the initial centre distribution approximated the profile of the confocal fluorophore image used for searching the fluorophores.

The time-gated pulsed STED E-PSF was approximated by a 2D Gaussian as defined by the quadratic increase in STED intensity near the minimum, the exponential depletion of the excited state and the detection PSF. A peak detection rate ε and a background detection rate β were assumed; that is a peak signal-to-background ratio $\mathrm{SBR} = \varepsilon/\beta$. The average detection rate $\bar{n}_i(t)$ at doughnut position $\vec{S}_i(t)$ was therefore

$$\bar{n}_i(t) = \varepsilon \exp\left(-4\ln(2)\frac{\left|\vec{S}_i(t) - \vec{r}_{\text{fl}}\right|^2}{d_{i-1}^2}\right) + \beta\,. \tag{S5}$$

The scan trajectory leading to the ith detection was determined by the centre position $\vec{C}_{i-1}$ and the radius R_{i-1} after the previous detection.

$$\vec{S}_i(t) = \vec{C}_{i-1} + R_{i-1}\begin{pmatrix}\cos(-\omega t)\\ \sin(-\omega t)\end{pmatrix} \tag{S6}$$

For each photon detection $i = 1 \ldots N$ an exponentially distributed number m_i with average value $\bar{m}_i = 1$ was drawn. The detection time t_i was then determined by integrating the signal $\bar{n}_i(t > t_{i-1})$ along the scan trajectory $\vec{S}_i(t)$ until its cumulative value reached m_i.

$$m_i = \int_{t_{i-1}}^{t_i} \bar{n}_i(t)\,\mathrm{d}t \tag{S7}$$

Upon each detection, the E-PSF was sharpened until reaching the minimal value d_{min} found for the maximal STED beam power. The scan radius was reduced equally until reaching its minimum R_{min}. Suppl. Fig. S4 illustrates that the doughnut position $\vec{S}_i(t)$ stays from the fluorophore at a narrow distance distribution centred on the scan radius during the entire localization. Therefore, the fluorophore is exposed only to a moderate STED intensity, which lowers photobleaching by the doughnut as compared to conventional raster-scanned STED imaging.

Camera-based localization uncertainty

The theoretical localization precision for single-molecule localizations by analysing camera images is given by Rieger and Stallinga[22] as

$$\sigma_{\text{cam}}^2 \approx \frac{\sigma_{\text{PSF}}^2 + p^2/12}{S}\left(1 + 4\tau + \sqrt{\frac{2\tau}{1+4\tau}}\right) \text{ with } \tau = \frac{2\pi b}{p^2 S}\left(\sigma_{\text{PSF}}^2 + \frac{p^2}{12}\right), \tag{S8}$$

where S is the total number of detected signal photons; p is the pixel size; b is the average background per pixel without dark counts; σ_{PSF} is the standard deviation of the image PSF approximated by a Gaussian spot; and σ_{cam} is the localization uncertainty.

We estimated the camera-based localization uncertainty for a pixel size of 100 nm and a diffraction-limited PSF at 670 nm wavelength. Fitting the simulated PSF with a 2D Gaussian profile yielded $\sigma_{\text{PSF}} = 116$ nm, whereas measuring the FWHM led to $\sigma_{\text{PSF}} = 118$ nm. Hence, we used $\sigma_{\text{PSF}} = 117$ nm. For the total image background B we considered 25 pixels: $B = 25b$. The total signal S is given by the peak signal s and the width of the 2D Gaussian distribution: $S = 2\pi s\,\sigma_{\text{PSF}}^2/p^2$. Hence, S was defined by the signal fraction of the detected photons $N = S + B$:

$$S = \frac{SN}{S+B} = \frac{N}{1 + \frac{25p^2}{2\pi\sigma_{\text{PSF}}^2}\frac{b}{s}} = \frac{N}{1 + \frac{2.91}{\text{SBR}}} \tag{S9}$$

With the chosen imaging parameters, equation (S8) evaluates to

$$\sigma_{\mathrm{cam}} \approx 121\ \mathrm{nm}\sqrt{\frac{1}{N}\left(1+\frac{2.91}{\mathrm{SBR}}\right)\left(1+\frac{4.24}{\mathrm{SBR}}+\sqrt{\frac{2.12}{\mathrm{SBR}+4.24}}\right)}. \qquad \text{(S10)}$$

The localization accuracy is further affected by any differences in pixel responses, which is particularly critical when seeking an uncertainty of 10 nm or less with (scientific) CMOS camera images.

Comparison of MINSTED and camera-based localization

Suppl. Fig. S1 illustrates the simulated localization uncertainty for different peak SBRs and the following settings: scan radius $R = d/2$ from 103 nm initially down to 13 nm; a centre update step $\alpha = 15$ % of the scan radius; a reduction factor $\gamma = 0.97$; $\omega = 2\pi \times 125$ kHz circling frequency; and $\varepsilon = 30$ kcps peak emission rate, corresponding to about 15–20 kcps average detection rate including background. The localizations were terminated after 10000 photon detections, or earlier when less than $n_{\mathrm{OFF}} = 10$ to 15 photons were detected in a $\tau_{\mathrm{OFF}} = 3$ ms interval. For each setting, we simulated 500 localizations. Less than 3% of the localizations terminated early. If the estimated fluorophore position $\hat{\vec{r}}_{\mathrm{fl}}(i)$ was further off than 5 times the median error of all simulated localizations, we deemed the localization as failure and excluded it for the remaining $i \dots N$ photon detections. A fraction of up to $1/\mathrm{SBR}$ localizations failed during the homing-in. An uncertainty of 1 nm along the x and y directions was obtained with 300 to 800 photon detections.

Suppl. Fig. S1 also shows the camera-based localization uncertainty without background and for the lowest peak SBR used in the simulations. Dotted lines extend these estimates for low photon numbers that are usually discarded. After a transitory phase, the scanning-based localization massively benefits from the zooming-in with an ever sharper E-PSF. Localization uncertainties of 3 nm or less can be achieved by camera-based localizations but require 30 to 50 times the number of detected photons than MINSTED.

If the fluorophore supports a higher exposure to STED light during the localization, a larger scan radius with respect to the PSF diameter further squeezes the required photon detections. Suppl. Fig. S2 shows that MINSTED can reach 1 nm precision with as few as 200 detected photons, which is about 50 times more photon-efficient than camera-based localizations and on par with iterative MINFLUX[8].

Comparison of MINSTED and MINFLUX

A detailed comparison with MINFLUX based on the achievable resolution is hampered by the numerous assumptions necessary for the different imaging parameters (e.g. fluorophore density, background, response of the background to STED). For a coarse comparison at large N, the localization precision of MINSTED can be approximated with a localization by two Gaussian beams placed at R distance on both sides of the molecule. Using this approximation, Suppl. Table T1 compares the major contributions to the performance of both methods.

Suppl. Table T1 | Estimated performance of MINFLUX and MINSTED.

	**MINFLUX**	**MINSTED**
**Fluorophore requirements**	High contrast between off and on state	High contrast between off and on state *under STED conditions*
**Resolution 1D (ref. 8)**	$\sigma = \frac{L}{4\sqrt{N}}$	$\sigma \approx \frac{\alpha d^2}{R\sqrt{N}} \ (\alpha < 1)$ Static Gaussian approximation: $\sigma = \frac{d^2}{4R \ln 2 \sqrt{N}}$
**Decreasing L/d with neighboring molecule**	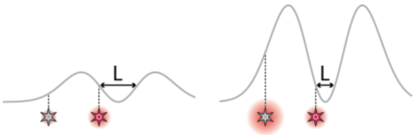 Undesired molecule is excited stronger than desired one	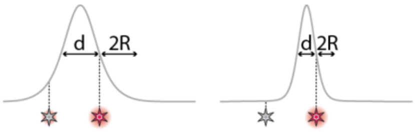 Undesired molecule is suppressed by STED
**Imperfect doughnut zero**	Additional counts are background counts → SBR decreases	Signal decreases → SBR decreases
**Excitable background**	Smaller L → stronger excitation of the background and weaker excitation of molecule → SBR decreases	Smaller d → same excitation of background and molecule → SBR constant
**STED-sensitive background**	-	Smaller d → background suppressed by stronger STED → SBR increases
**STED-induced background (not observed)**	-	Smaller d → more background by stronger STED → SBR decreases

Animations

Suppl. Video V1 illustrates the evolutions of the centre-to-fluorophore distance distributions during the detection of 100 photons for update steps $\alpha = 10\%$, 15%, 20% and 25% of the scan radius and for peak SBR of 50, 20, 10, 5 and 2. The diameter of the solid circle and the radius of the dashed circle equal the E-PSF diameter d, which is twice the scan radius R. Each run starts with a uniform centre-to-fluorophore distance distribution in the dashed circle. If the centre-to-fluorophore distance leaves this region, the fluorophore is considered "lost" because the centre would rarely re-approach the fluorophore in practice. Fluorophores get lost mostly during the first few detections when a step in the wrong direction can be fatal. Once the distribution converges, losses occur only if the background is too high and/or the step too large. Towards the end of each run, the standard deviation σ_C of the converged distribution is shown in units of the scan radius.

Suppl. Video V2 animates the localization of a fluorophore. Yellow to red dots mark the most recent centre coordinates and the circular line illustrates the recent doughnut positions. Photon detections are illustrated by a flash with the shape of the E-PSF at the doughnut position of the detection event. The scan trajectory is updated immediately upon the detection of a photon. Weighted histograms of the centre positions are shown above and to the right of the image. After homing in on the

fluorophore, these histograms converge towards normal distributions, whose centres indicate the fluorophore position and whose standard deviations equal the values shown in Suppl. Video V1. The animation is sped up about ten-fold by removing full scan circles without detection event.

MINSTED microscope

The core of the implemented MINSTED microscope is outlined in Suppl. Fig. S7. The microscope incorporates a laser beam-scanning path and descanned confocal detection using two galvo scanners to address a field of $100\ \mu m \times 100\ \mu m$ in the sample. A cw laser with 633 nm wavelength provides the excitation light and a single-photon avalanche diode (APD) detects the fluorescence light emitted from the sample. A 355 nm cw laser beam illuminates a central region of about 4 µm diameter to activate the fluorophores. These two laser beams are s-polarized on the main dichroic mirror DM_2 that was customized to transmit the p-polarized laser beams from the second excitation path with pulsed 635 nm excitation light and 775 nm STED light. The STED beam passes through a vortex plate VP to create the lateral STED doughnut. A purposefully tilted notch filter N cleans up its polarization and an achromatic quarter-wave retardation plate (λ/4) sets up the required circular polarization. The second excitation path features two electro-optical deflectors (EODs) to address rapidly a field of $2.6\ \mu m \times 2.6\ \mu m$ without any mechanical movement. The control electronics and the driver of the EODs provide a bandwidth of 150 kHz, which is used to scan the beam in circles around the estimated position of the fluorophore at 125 kHz frequency.

A two-axis stick-and-slip piezo stage moves the sample laterally over large distances, such that the region of interest can be centred to the EODs' image field. The sample position is actively stabilized by a three-axis piezo stage with sub-nanometre precision. The focus feedback signal is obtained by tracking the position of the reflected beam on the z-lock camera CAM_z (out-of-plane excursion in Suppl. Fig. S7). The lateral position feedback signal is obtained by tracking the images of fiducial markers on the x-y-lock camera CAM_{xy}. The fiducial markers are imaged in a field of about $50\ \mu m \times 70\ \mu m$ off-axis to avoid interference with the imaged field of the sample. The tracker field is limited by the field stop F. Both focus locks are polarization filtered to suppress stray light and reflections as much as possible. For the x-y-lock, a pupil filter P is used to block the direct reflection at the coverslip–sample interface. Infrared filters IR block the excitation and STED light below 850 nm wavelength.

The z-lock uses an 8-bit CMOS camera imaging the lateral position of the reflected beam at 800 to 1500 frames per second (fps) depending on the extent of the selected region of interest. 16 consecutive camera images are binned and then processed to extract the beam centre. The deviation of the beam centre with respect to the target position is integrated and scaled to obtain the control signal. Including mechanical inertia, the closed-loop control bandwidth was 15 to 30 Hz.

The x-y-lock uses a 16-bit sCMOS camera imaging fiducials with 150 to 200 fps depending on the extent of the region of interest. The fiducial positions are estimated by least squares fitting of their images to a two-dimensional Gaussian profile with constant background. The deviation of the lateral positions of trustworthy fiducials with respect to their initial positions is integrated and scaled to obtain the control signal. The closed-loop control bandwidth was about 80 Hz. Fiducials are manually selected as trustworthy if neither their positions nor their intensities fluctuated noticeably.

For clarity, the polarization, spatial and spectral cleaning of the laser beams and their power modulations are not shown. In particular, the APD, the lasers at 355 and 775 nm wavelength and the super-luminescent LED at 980 nm wavelength are pig-tailed and/or fibre-coupled to the system. The excitation lasers at 633 and 635 nm wavelengths are fed through pinholes to clean-up the beam profiles. All laser beams are linearly polarized by Glan-Thompson polarizers (Bernhard Halle) and

spectrally cleaned by excitation filters (Chroma Technology and Semrock). All light sources can be blocked by shutters and their beam powers can be modulated internally or externally.

Main components

355nm	Zouk 0355-05-01-0010-500 (10 mW cw), Cobolt, Solna, Sweden; beam power modulated by an acousto-optic modulator MQ110-A3-UV with driver MODxx, AA optoelectronic, Orsay, France; fed through polarization-maintaining single-mode fibre PMC-360Si-2,3-NA012-3-APC-500P, Schäfter+Kirchhoff, Hamburg, Germany.
633nm	HeNe laser 25-LHP-073-230 (< 25 mW cw), Melles Griot, Rochester, NY, USA.
635nm	Pulsed diode laser LDH-P-C 635b (< 80 MHz, < 100 ps, < 1 mW) with driver PDL 800-B, PicoQuant, Berlin, Germany.
775nm	Pulsed fibre laser ELP-5-755-DG (20 MHz, 1.2 ns, < 5 W), IPG Photonics, Oxford, MA, USA; beam power modulated by an electro-optic modulator LM0202 5W VIS, Linos Photonics, Göttingen, Germany; with custom driver 400 V, 75 mA, 600 kHz, MPI for Biophysical Chemistry, Göttingen, Germany; fed through polarization-maintaining single-mode fibre PM780-HP-FC/APC, Thorlabs, Newton, NJ, USA.
980nm	Super-luminescent LED SLD-1000-100-PM-25, Innolume, Dortmund, Germany; with driver LDR1000E, Laser 2000, Wessling, Germany.
APD	Single-photon counting module SPCM-AQR-13-FC, PerkinElmer, Wiesbaden, Germany.
CAM_{xy}	USB3 sCMOS camera pco.panda 4.2 (2048×2048 pixels 6.5×6.5 μm^2, 16 bits, 100 fps), PCO, Kelheim, Germany.
CAM_z	USB3 CMOS camera acA1300-200um (1280×1024 pixels 4.8×4.8 μm^2, 8/10 bits, 203 fps), Basler, Ahrensburg, Germany.
EOD	Electro-optic deflectors 311A (AD*P, Ø2 mm, 200 mm long, 7 µrad/V, 180 pF, ±500 V), Conoptics, Danbury, CT, USA; high-voltage drivers PZD700A-1-H-SHV-CE (±700 V, 200 mA, 150 kHz), Trek, Lockport, NY, USA.
FPGA	PCIe board 7852R with drivers and software LabVIEW 2017, National Instruments, Austin, TX, USA.
$Galvo_{x,y}$	Galvo-mirrors 6 mm × 10 mm, galvo 6215H, servo drivers 671, Cambridge Technology, Bedford, MA, USA.
Objective	Magnification 100×/1.4 NA oil-immersion, Leica Mikrosysteme, Wetzlar, Germany.
Stages	Three-axis piezo stage P-733.3DD with controller E-725.3CDA on two-axis piezo stage M-686.D64 with controller C-867.262, Physik Instrumente, Karlsruhe, Germany.

Filters

F	Adjustable rectangular field stop
P	Custom pupil filter Ø6.5 mm with central obscuration Ø1.4 mm on 2 mm thick window.
PH	Pinhole Ø300 µm
VP	Vortex plate VL-209-M-Y-A, Holo-Or, Ness Ziona, Israel.
DM_1	Dichroic mirror Z660DCXR, Chroma Technology, Bellows Falls, VT, USA.
DM_2	Custom dichroic mirror HR350-730nm HT450-475nm/p+630-640nm/p at 45° incidence, LaserOptik GmbH, Garbsen, Germany.
DM_3	Back-polished mirror BB01-E02P, Thorlabs, Newton, NJ, USA.
DM_4	Dichroic mirror Z355RDC, Chroma Technology, Bellows Falls, VT, USA.
DM_5	Notch filter StopLine NF01-633-25, Semrock, Rochester, NY, USA.
E	Emission filters ET750-SP-2P8 and ET680/80m, Chroma Technology, Bellows Falls, VT, USA.
IR	Infrared filter RG 850 (2 mm thick), Schott, Mainz, Germany.
N	Notch filter StopLine NF03-808E-25, Semrock, Rochester, NY, USA.

Lenses

L_1	Achromatic doublet 200 mm, 322328000, Linos Photonics, Göttingen, Germany.
L_2	Achromatic doublet 40 mm, AC254-040-B, Thorlabs, Newton, NJ, USA.
$L_{3,8,10,14}$	Achromatic doublet 300 mm, 322273322, Linos Photonics, Göttingen, Germany.
$L_{4,5,6}$	Achromatic doublets 100 mm, 49-333, Edmund Optics, Mainz, Germany.
L_7	Achromatic doublet 60 mm, 49-329, Edmund Optics, Mainz, Germany.
L_9	Asphere 30 mm, 355 nm V-coating, 33-012, Edmund Optics, Mainz, Germany.
$L_{11,12,13}$	Hasting triplets 40 mm, THR254-040-A, Thorlabs, Newton, NJ, USA.
$L_{15,19}$	Achromatic doublet 160 mm, 322270322, Linos Photonics, Göttingen, Germany.
L_{16}	Achromatic doublet 16 mm, 322207000, Linos Photonics, Göttingen, Germany.
L_{17}	Fibre collimator 11 mm, 60FC-0-A11-02, Schäfter+Kirchhoff, Hamburg, Germany.
L_{18}	Achromatic doublet 40 mm, 322337000, Linos Photonics, Göttingen, Germany.
L_{20}	Achromatic doublet 100 mm, AC254-100-B, Thorlabs, Newton, NJ, USA.
L_{21}	Achromatic doublet 400 mm, 322275322, Linos Photonics, Göttingen, Germany.
L_{22}	Achromatic doublet 200 mm, 322353000, Linos Photonics, Göttingen, Germany.

Polarization optics

GT	Glan-Thompson polarizing prisms PGT 1.08, Bernhard Halle, Berlin, Germany.
PBS	Polarizing beam splitter cubes PTW 2.20, Bernhard Halle, Berlin, Germany.
λ/4	Achromatic quarter-wave retarder plate RAC 4.4.10, Bernhard Halle, Berlin, Germany.
λ/2	Achromatic half-wave retarder plates RAC 4.2.10, Bernhard Halle, Berlin, Germany.

Supplementary references

22. Rieger, B. & Stallinga S. The Lateral and Axial Localization Uncertainty in Super-Resolution Light Microscopy. *ChemPhysChem* **15**, 664–670 (2014).

Supplementary figures

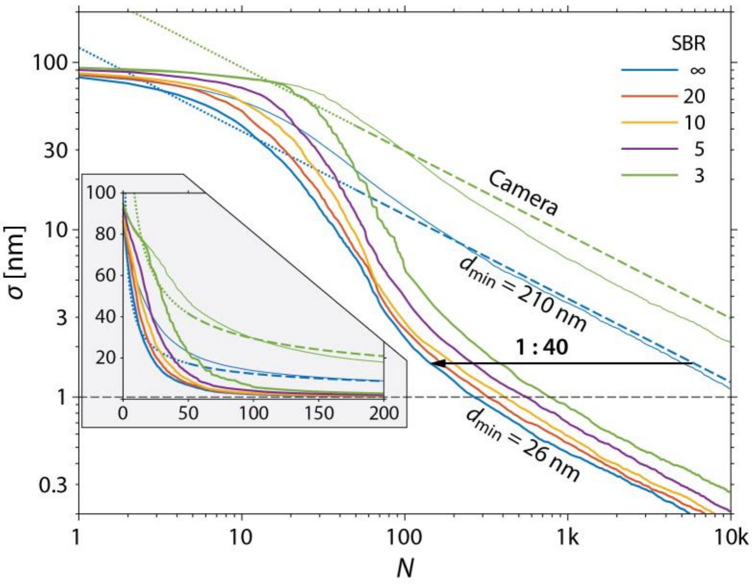

Suppl. Fig. S1 | Simulated localization uncertainty versus number of detected photons and SBR. The localizations were performed by zooming-in from $d_0 = 210$ nm PSF diameter and $R_0 = 103$ nm scan radius down to $d_{\min} = 26$ nm and $R_{\min} = 13$ nm. For $\mathrm{SRB} = \infty$ and 3, dashed and dotted lines show the camera-based CRBs and thin lines show the uncertainties with diffraction-limited zooming-in on the fluorophore.

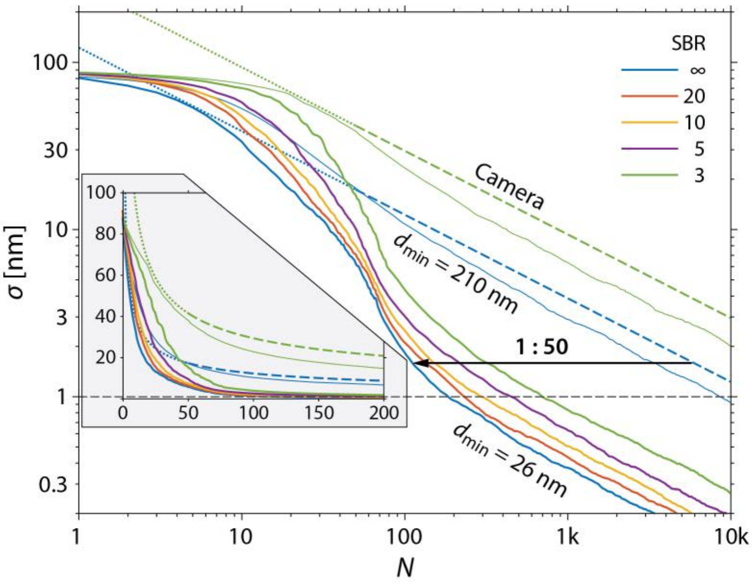

Suppl. Fig. S2 | Simulated localization uncertainty versus number of detected photons and SBR. The localizations were performed by zooming-in on the fluorophore from $d_0 = 210$ nm PSF diameter and $R_0 = 130$ nm scan radius down to $d_{\min} = 26$ nm and $R_{\min} = 17$ nm. The inset shows the results in linear scale for small photon numbers. For $\mathrm{SRB} = \infty$ and 3, dashed and dotted lines show the camera-based CRBs and thin lines show the uncertainties with diffraction-limited zooming-in.

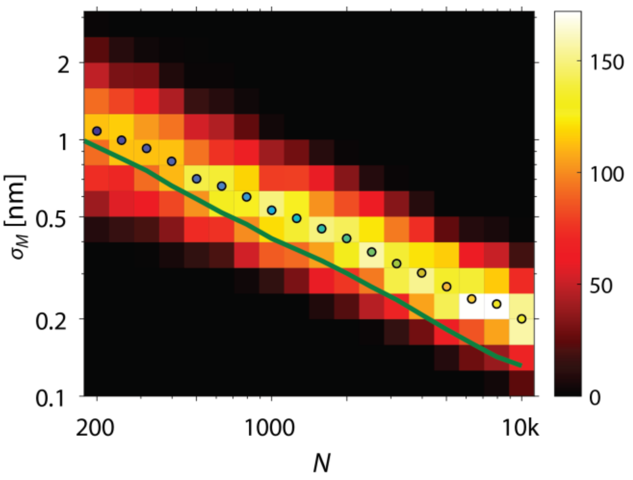

Suppl. Fig. S3 | Simulated versus estimated localization precision. The simulated localization uncertainty (solid line) was obtained by Eq. $(S2)$ and the estimated localization precision (histogram; dots: median) by Eq. $(S4)$.

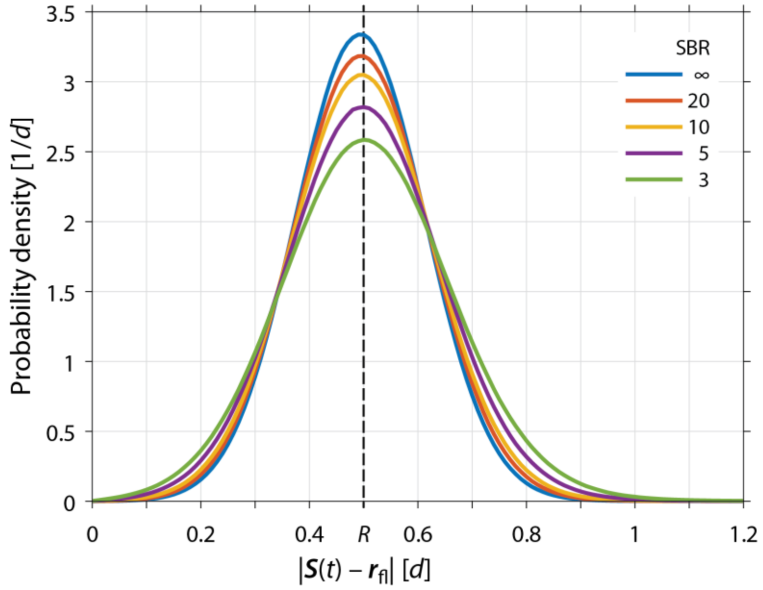

Suppl. Fig. S4 | Distribution of the doughnut-to-fluorophore distances during the simulated localizations of Suppl. Fig. S1.

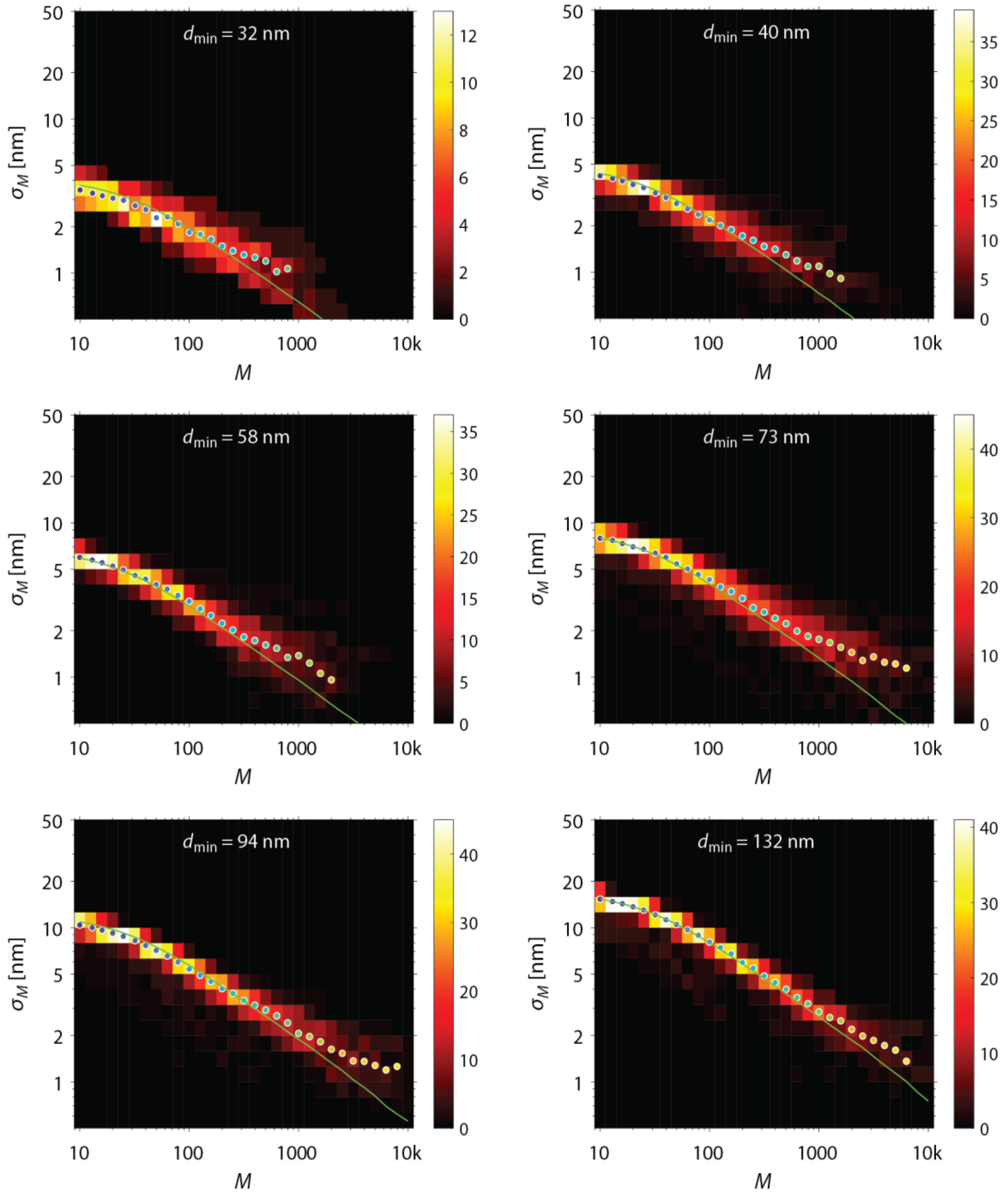

Suppl. Fig. S5 | Localization precision measurements versus E-PSF diameter d_{min}, see also Fig. 3c. Localization precision histograms of grouped localization traces of single molecules and their median localization precision (dots) compared to simulations (lines).). The simulations assumed an SBR of 10 for $d_{\mathrm{min}} = 32$, 94 and 132 nm, an SBR of 20 for $d_{\mathrm{min}} = 40$ and 73 nm and infinite for $d_{\mathrm{min}} = 58$ nm.

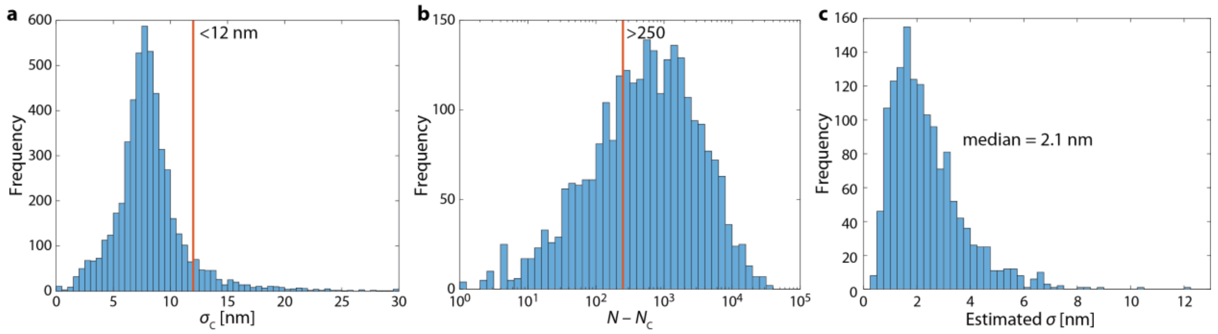

Suppl. Fig. S6 | Characteristics of the localizations measured for Fig. 4. a, Distribution of the standard deviation of the centre positions. **b**, Distribution of the number of detected photons used for estimating the fluorophore position. **c**, Distribution of the estimated localization precision of the rendered localizations.

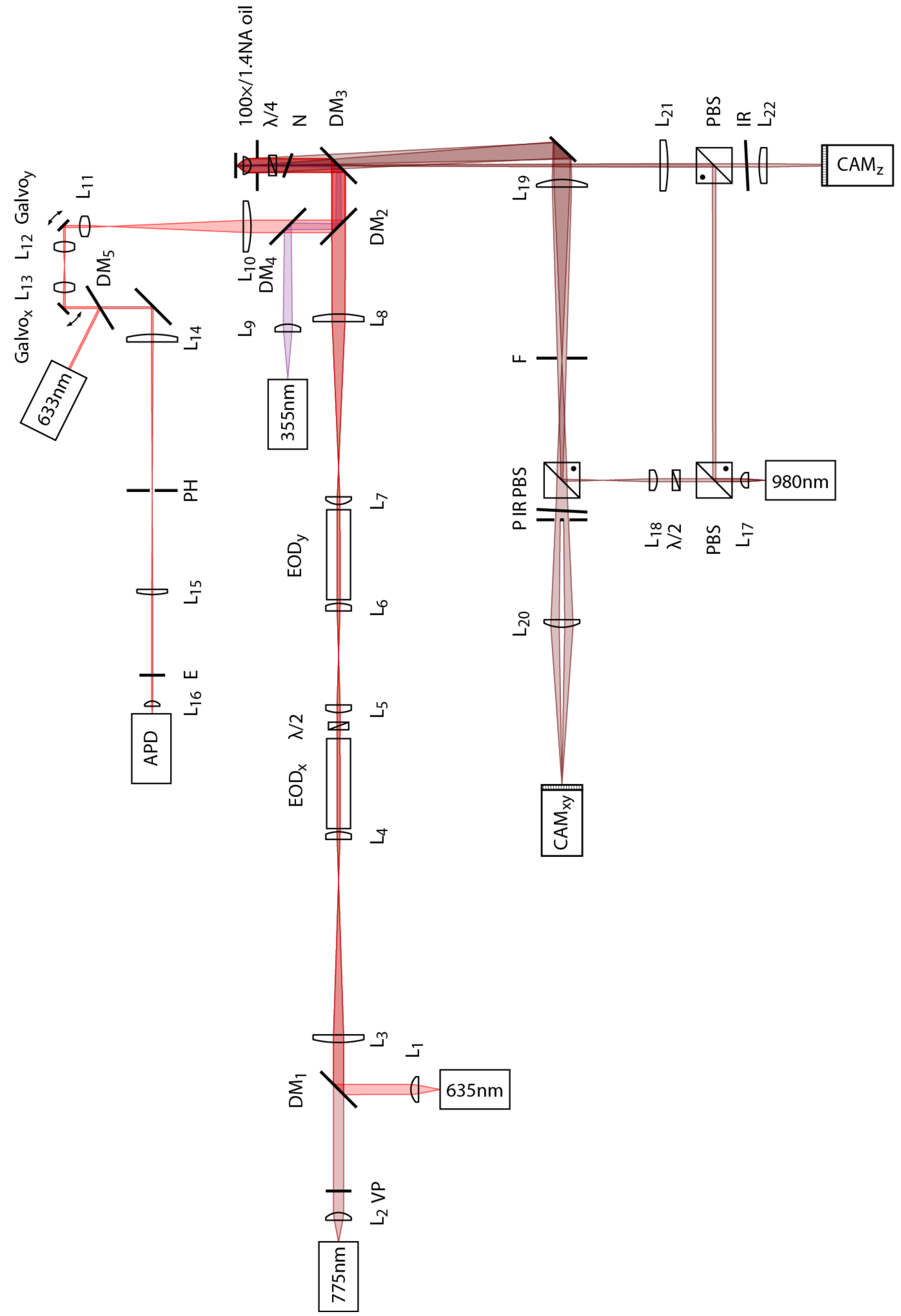

Suppl. Fig. S7 | Optical scheme of the MINSTED microscope. The lasers with 355 and 775 nm wavelength, the super-luminescent LED at 980 nm wavelength and the APD are fiber-coupled to the setup. All light sources are controlled by shutters and their powers are modulated or attenuated. The sample position is actively stabilized by feedback signals from the Z and X-Y focus locks.

Supplementary videos

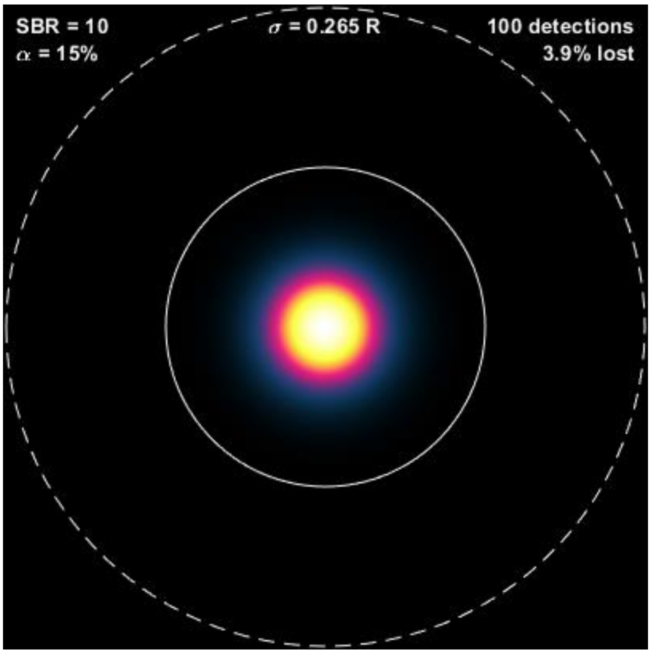

Suppl. Video V1 | Distributions of the centre-to-fluorophore distances during the detection of 100 photons while circling with constant scan radius and constant E-PSF. The solid circle illustrates the scan trajectory and the E-PSF diameter.

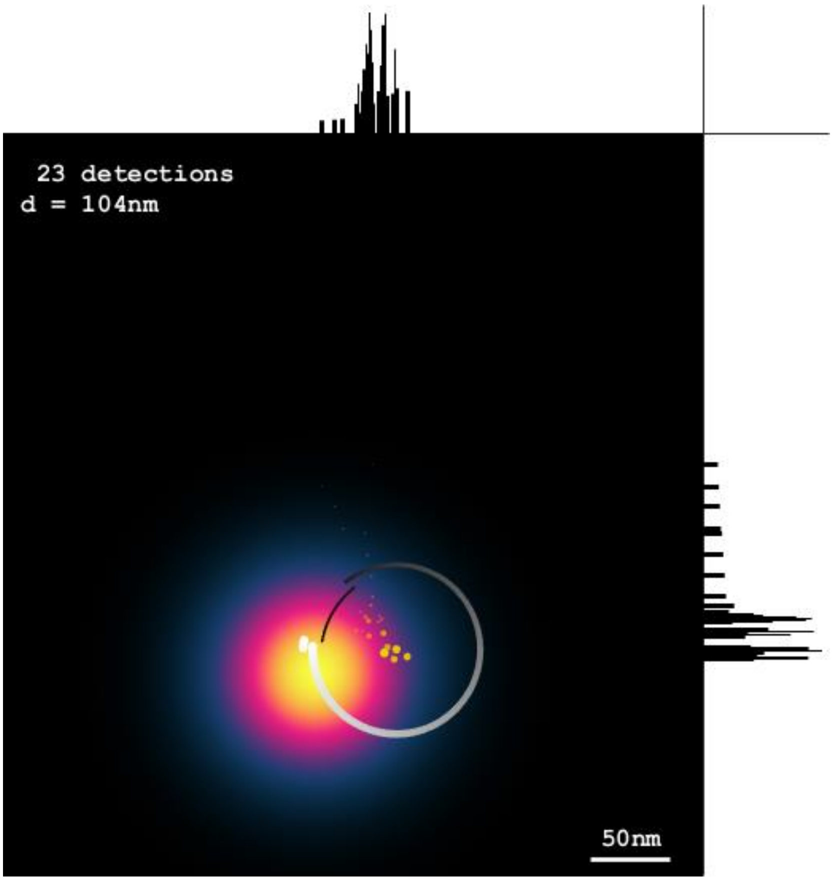

Suppl. Video V2 | Animation of the localization of a single fluorophore with typical settings: scan radius $R = \mathrm{d}/2$, update steps $\alpha = 15\%$ and $\gamma = 0.97$.
